# Thinking beyond the tablet: Acceptability of different HIV-PrEP modalities among GBMSM and gender-diverse individuals having sex with men in the UK

**DOI:** 10.1177/09564624251369093

**Published:** 2025-08-22

**Authors:** Dana Ogaz, Natasha Ratna, Hridhya Vijayan, Stephanie J Migchelsen, David Reid, Dolores Mullen, Dawn Phillips, Eleanor Bell, Tamara Djuretic, Will Nutland, Catherine H Mercer, Kate Folkard, John Saunders, Hamish Mohammed

**Affiliations:** 1 Blood Safety, Hepatitis, STI & HIV Division, 371011 UK Health Security Agency, London, UK; 2 The National Institute for Health and Care Research Health Protection Research Unit in Blood Borne and Sexually Transmitted Infections at University College London in Partnership with the UK Health Security Agency, London, UK; 3 Institute for Global Health, 4919 University College London, London, UK; 4 The Love Tank, London, UK

**Keywords:** HIV (Human immunodeficiency virus), viral disease, prevention, other, homosexual, other, men, other

## Abstract

**Background:**

HIV-PrEP is a key component of HIV combination prevention and has been routinely available through oral formulations (i.e. oral tablets) across sexual health services (SHSs) in the UK since 2020. We used data from a large, online community survey to assess the acceptability and preference of different HIV-PrEP modalities among gay, bisexual, and other men who have sex with men (GBMSM) and gender-diverse individuals living in the UK.

**Methods:**

Using data collected from the ‘Reducing inequalities in Sexual Health’ (RiiSH) survey, an online community survey of 1106 GBMSM and gender-diverse individuals having sex with men (November/December 2023), we performed a secondary analysis examining HIV-PrEP modality acceptability and preference (e.g. oral, long acting injectable, gel, or patch) (%) by HIV-PrEP history (never, in lookback period of last 3–4 months since survey completion). Where ≥2 modalities were indicated, participants were asked to specify a single preferred modality. Single choice options were assumed to be the preferred HIV-PrEP modality.

**Results:**

Irrespective of HIV-PrEP history, long acting injectables were highly acceptable (>70%) and the preferred modality (58% [222/386] by those never having used HIV-PrEP, 73% [75/103] in those without HIV-PrEP use in the lookback, 74% [314/424] in those with HIV-PrEP use in the lookback).

**Conclusions:**

We found high acceptability of the use of injectables, with general preference over oral tablets. If injectable HIV-PrEP becomes available at SHSs in England, these modalities could have positive impacts on HIV-PrEP delivery, use, and access.

## Background

In England, HIV disproportionately affects gay, bisexual, and men who have sex with men (GBMSM), who represented nearly a third of new HIV diagnoses in 2023 despite comprising a minority (<5%) of the population.^1,2^ Alongside scale-up of repeat HIV testing and immediate antiretroviral treatment (ART) as prevention,^3,4^ HIV-PrEP comprises a key component of HIV combination prevention. Since the start of routine commissioning in 2020, over 80,000 individuals, primarily GBMSM, have accessed HIV-PrEP through sexual health services (SHSs) in England.^1^ At present, HIV-PrEP is dispensed across SHSs in tablet form for daily or event-driven use, with quarterly (or at least 6-monthly) testing for HIV and STIs recommended.^5^ While long acting injectable formulations of cabotegravir and lenacapavir have shown promise as alternatives to tablet-based formulations for cisgender men, cisgender and transgender women,^6–8^ recommendations for use as HIV-PrEP across publicly funded SHSs in the UK is currently under review.^9^ To date, there are limited data on the acceptability of different HIV-PrEP modalities among GBMSM in the UK. We use data collected from RiiSH 2023, an online community survey, to assess the acceptability and preference of new HIV-PrEP modalities, including those not yet available through routine commissioning.

## Methods

### Data collection and recruitment

The ‘Reducing inequalities in Sexual Heath’ (RiiSH) surveys, a series of online cross-sectional surveys of a community sample UK-resident GBMSM and gender-diverse individuals having sex with men have been implemented annually to inform the sexual health and well-being of participants and used to supplement national STI surveillance where behavioural data is limited.^10–12^ RiiSH survey methods have been previously reported.^10^ Recruitment for RiiSH 2023 took place from 7 November to 6 December 2023 through social networking sites (Facebook, Instagram, X) and a geospatial dating application (Grindr). Self-identifying men (either cisgender or transgender), transgender women, or gender-diverse (nonbinary) individuals assigned male at birth, aged ≥16 years, resident in the UK, and reporting sex with a man in the last year were able to take part. RiiSH survey data was collected via the Snap Surveys platform (https://www.snapsurveys.com/). Data management and analyses were conducted using Stata v17.0 or higher (StataCorp, College Station, TX, USA).

### HIV-PrEP modality acceptability and preference among RiiSH 2023 participants

We performed a secondary analysis to assess the acceptability of new HIV-PrEP modalities reported among participants when asked if they would consider use of a series of HIV-PrEP modalities, including (already available) oral tablets, assuming similar levels of efficacy (see Table 1 for modality selections and survey questions). We report all modalities considered by participants (%) and their preferred modality by HIV-PrEP history (i.e., never used, ever use outside of preceding 3–4 months [i.e. lookback period], ever use in lookback period [mutually exclusive]) (Table 1). Where two or more modalities were considered by a participant, a follow-up question asked participants to specify a single preferred modality. Single choice options were assumed to be the preferred modality. Those specifying ‘none’ for considered modalities were not routed to the follow-up question.

**Table 1.** HIV-PrEP modality acceptability and preference reported by RiiSH 2023 participants.

a) All considered HIV-PrEP modalities (i.e., acceptability)^a^			
	Never used HIV-PrEP^b^, *n* (%)	HIV-PrEP use outside of lookback^b^, *n* (%)	HIV-PrEP use in lookback^b^, *n* (%)
RiiSH 2023 participants (*n* = 963)^c^	433 (100%)	105 (100%)	425 (100%)
None of these (exclusive)	47 (11%)	<5 (<3%)^d^	<5 (<3%)^d^
Long acting injectable that lasts up to 2 months	12 (29%)	49 (47%)	214 (50%)
Long acting injectable that lasts up to 6 months	206 (48%)	66 (63%)	309 (73%)
Long acting injectable that lasts a year or more	283 (65%)	86 (82%)	336 (79%)
Any long acting injectable	*307 (71%)*	*91 (87%)*	*376 (88%)*
Removable implant	75 (17%)	25 (24%)	116 (27%)
Adhesive skin patch	138 (32%)	33 (31%)	124 (29%)
A gel, cream or foam inserted in the rectum	81 (19%)	27 (26%)	75 (18%)
Oral tablets^e^	209 (67%)	71 (68%)	327 (77%)

b) Preferred modalities^f^			
	Never used HIV-PrEP^g^, *n* (%)	HIV-PrEP use outside of lookback^g^, *n* (%)	HIV-PrEP use in lookback^g^, *n* (%)
RiiSH 2023 participants who considered one or more HIV-PrEP modality^h^	386 (100%)	103 (100%)	424 (100%)
Long acting injectable that lasts up to 2 months	<10 (<3%)^i^	<5 (<5%)^i^	16 (4%)
Long acting injectable that lasts up to 6 months	<35 (<10%)^i^	<15 (<15%)^i^	76 (18%)
Long acting injectable that lasts a year or more	182 (47%)	*58 (56%)*	222 (52%)
Any long acting injectable	*222 (58%)*	*75 (73%)*	*314 (74%)*
Removable implant	<10 (<3%)^i^	<5 (<5%)^i^	10 (2%)
Adhesive skin patch	22 (6%)	5 (5%)	<10 (<3%)^i^
A gel, cream or foam inserted in the rectum	<10 (<3%)^i^	<5 (<5%)^i^	<10 (<3%)^i^
Oral tablets^j^	129 (33%)	22 (21%)	87 (21%)

^a^Included in survey “Note, HIV-PrEP tablets are already available and highly effective at preventing HIV when taken as prescribed”.

^b^HIV-PrEP history categories are mutually exclusive. Note, modality responses are not mutually exclusive. HIV-PrEP: HIV pre-exposure prophylaxis. RiiSH: ‘Reducing inequalities in Sexual Health’.

^c^Among all participants, aged ≥16, with a HIV-negative or unknown status.

^d^Suppressed due to small numbers.

^e^Survey question: “New forms of HIV PrEP are in development. If all were available and all worked equally well at preventing HIV, which form(s) would you consider using (tick all that apply)”. HIV-PrEP use outside of lookback: report of use outside of last 3–4 months (i.e. before August 2023). HIV-PrEP use in lookback: report of use in last 3–4 months (i.e. since August 2023).

^f^Included in survey “Note, HIV-PrEP tablets are already available and highly effective at preventing HIV when taken as prescribed”.

^g^HIV-PrEP history categories are mutually exclusive. Note, modality responses are mutually exclusive and restricted to those who considered one or more modality (see 1a).

^h^Among all participants, aged ≥16, with a HIV-negative or unknown status.

^i^Suppressed due to small numbers.

^j^Survey question: “New forms of HIV PrEP are in development. If all were available and all worked equally well at preventing HIV, which form(s) would you consider using (tick all that apply)”. Follow on question (where two or more responses specified): “Based on your previous choices, which forms of HIV PrEP would you consider using most (i.e., which would you use first)”. HIV-PrEP use outside of lookback: report of use outside of last 3–4 months (i.e. before August 2023). HIV-PrEP use in lookback: report of use in last 3–4 months (i.e. since August 2023).

Using Pearson’s χ^2^ test, we assessed differences (where p < .05) in preferred modality by sociodemographic characteristics (age group, ethnicity, country of birth, financial stability) and behavioural characteristics including ≥5 male condomless anal sex partners in the lookback period. For these analyses, we grouped modalities as (1) any long acting injectable (which included 2-monthly, 6-monthly and yearly formulations), (2) oral tablets, and (3) other modalities (which included removable implant, adhesive skin patch, a gel, cream or foam).

## Ethics statement

Ethical approval for RiiSH 2023 was granted by the UKHSA Research and Ethics Governance Group (REGG; ref: R&D 524). Online consent was collected from participants and there was no incentive offered to participate.

## Results

There were 1,106 participants who were eligible to take part in RiiSH 2023, of which, 963 were HIV-negative or of unknown HIV status (hereafter HIV-negative) and included in analyses. The median age of included participants was 43 years (interquartile range: 33–53). Most were of White ethnicity (89%, 859/963), cisgender male (95%, 915/963), degree-educated (63%, 607/963), employed (78%, 751/963), and England residents (84%, 813/963). Two-in-five reported financial stability (i.e. comfortable/very comfortable financial situation) (42%, 404/963) and most were UK born (78%, 747/963). Over two-thirds (65%, 622/963) of participants that were HIV-negative reported condomless anal sex with a male partner in the lookback period (last 3–4 months), of which 29% (179/622) reported ≥5 partners.

### HIV-PrEP modality acceptability and preference among RiiSH 2023 participants

Nearly half of HIV-negative participants reported never having used HIV-PrEP (45% 433/963). Of those ever reporting HIV-PrEP use (55% 530/963), most reported use in the lookback period (80% 425/530). Irrespective of HIV-PrEP history, long acting injectables were highly acceptable (>70%; Table 1a) and the preferred modality (58% [222/386] in those never having used HIV-PrEP, 73% [75/103] in those without HIV-PrEP use in the lookback, 74% [314/424] in those with HIV-PrEP use in the lookback, Table 1b).

We found no differences in modality preference by sociodemographic characteristics, but we found higher preference of any long acting injectable in those reporting ≥5 male condomless anal sex partners (76% vs 65% in those with <5 partners) (Table 2).

**Table 2.** Preferred HIV-PrEP modalities by sociodemographic and behavioural characteristics reported by RiiSH 2023 participants.

	Preferred HIV-PrEP modality			χ^2^ p-value
	Any long acting injectable	Tablet	Other form	
	Row %	Row %	Row %	
Total	67% (611)	26% (238)	7% (64)	
Sociodemographic characteristics				
Age group				
16–29	60% (85)	30% (41)	10% (14)	
30–44	70% (251)	23% (83)	6% (21)	
≥45	66% (275)	28% (114)	7% (29)	0.214
Ethnicity				
White	67% (544)	26% (214)	7% (55)	
Other ethnicities	67% (67)	24% (24)	9% (9)	0.664
UK-born				
Yes	66% (467)	26% (189)	7% (49)	
No	69% (144)	24% (49)	7% (15)	0.644
Comfortable financial situation				
Yes	68% (258)	26% (100)	6% (21)	
No	66% (352)	26% (138)	8% (43)	0.332
Behavioural characteristics				
Condomless anal sex with a man in the lookback period				
Yes	69% (417)	24% (144)	6% (37)	
No	62% (194)	30% (94)	9% (27)	0.042
≥ 5 condomless anal sex partners in the lookback period				
Yes	76% (133)	18% (32)	6% (11)	
No	65% (478)	28% (206)	7% (53)	0.020

*Based on specified responses for following questions: (1) “New forms of HIV PrEP are in development. If all were available and all worked equally well at preventing HIV, which form(s) would you consider using (tick all that apply)”; (2) Follow on question (where two or more responses specified): “Based on your previous choices, which forms of HIV PrEP would you consider using most (i.e., which would you use first). Any long acting injectable = long acting injectable lasting up to 2 months, 6 months, or a year or more (see Table 1); Tablet = oral tablets (see Table 1). Other form = removable implant, adhesive skin patch, a gel, cream or foam inserted in the rectum (see Table 1).

## Discussion

We assessed the acceptability of new HIV-PrEP modalities among GBMSM in the UK, where HIV-PrEP has been available in all UK nations since 2020. Among our community sample of GBMSM, we found high acceptability of long acting injectables, with general preference over oral tablets. Findings can inform HIV-PrEP delivery strategies aiming to promote accessibility and minimise health inequalities as HIV-PrEP availability through SHSs and other settings continues to evolve.

Our findings corroborate international studies and affirm high acceptability of long acting injectables alongside tablet formulations among those who have, and have not, ever used HIV-PrEP. Similar to North and Latin American studies,^13,14^ we found differing modality preference by markers of sexual risk, as those with higher numbers of condomless anal sex partners reported preference for long acting injectables relative to oral tablets. Among groups who never used HIV-PrEP, we found similar, and high acceptability for both oral tablets (67%) and any long acting injectable (71%), though we have no indication as to why these groups may have never used HIV-PrEP (note 10% [41/433] had tried and were unable to get HIV-PrEP in the lookback period, not shown). An Australian study of GBMSM using HIV-PrEP^15^ reported high acceptability and preference for long acting modalities compared to daily tablets, partly due to concerns about side effects and self-perceived adherence difficulties.^16^ This may drive similar differences in our study sample, as we see higher magnitudes of long acting injectable acceptability (>85%) and overall preference relative to oral tablets among recent and ever HIV-PrEP users.

Our study posited questions hypothetically assuming equal efficacy among all modalities, as well as considerations of use for long acting modalities not yet routinely available in the UK (e.g. 2-monthly, 6-monthly, and yearly formulations). However, evidence for the use of long acting injectables for HIV prevention among GBMSM has been promising. Randomised control evidence has demonstrated lower HIV incidence and fewer adverse drug reasons among those receiving 2-monthly injections of long acting cabotegravir compared to daily oral HIV-PrEP,^7^ which could improve adherence and further reduce risk of HIV. Among men and gender-diverse persons having sex with men, HIV incidence was lower among those with twice-year injections (i.e. 6-monthly) of lenacapavir relative to background HIV incidence. Though acceptability and preference for long acting injectables was high in our sample, and increased with the length of proposed effectiveness, this is not an indication of future use. Our study sample may not be generalisable to the wider population of UK GBMSM and may represent a highly health literate, and socially mobile group.^17,18^ Like many convenience samples, RiiSH data is subject to selection bias, however, provide expanded insights to sexual risk behaviours and partnerships, as well as crucial information among groups not accessing SHSs. While we found no sociodemographic differences in preference of HIV-PrEP modalities, we are limited by the RiiSH 2023 sample size which may limit inferences across groups and between nations, though most participants resided in England. However, we generally see higher preference for long acting injectables among those with greater markers of sexual risk (e.g. ≥5 condomless anal sex partnerships, those currently using HIV-PrEP), which is consistent with other studies and indicates the utility of alternative modalities in future HIV-PrEP delivery strategies. Among those reporting HIV-PrEP use in the lookback, we did not assess preference by most current HIV-PrEP regimen (e.g. daily or event-based), which could have influenced responses due to the differing pill burden between daily and less frequent use.

### Implications

Our findings suggest that among a community sample of GBMSM in the UK, there is high acceptability for other HIV-PrEP modalities outside of already available and highly effective oral tablets. At present, longer acting formulations as alternatives to daily formulations are currently being assessed by the National Institute for Health and Care Excellence (NICE) in the UK.^9^ The availability of HIV-PrEP through free and open-access SHSs in England, presents opportunities and unique implementation challenges that may differ from those in other high-incomes settings.^19^ Integration of long acting formulations raises a range of service-level challenges, including the need for enhanced clinical resourcing (e.g. training and education), adaptions to service delivery, and the need for tailored health promotion strategies. On the other hand, uptake of long acting injectables could release scarce clinic capacity which could be directed towards improving oral and injectable uptake for other groups with HIV-PrEP need, including heterosexual men and women,^1^ and free resource for complex clinical cases. While alternative modalities have the potential to address persistent access barriers and social inequalities that have emerged through routine availability of oral HIV-PrEP, such as stigma and knowledge barriers,^20,21^ there remains limited understanding of individual-level knowledge needs for those who could benefit from HIV-PrEP, and equally importantly, service-level implications these could have on HIV-PrEP care continuums. If injectable formulations for HIV-PrEP are introduced in SHSs, it will be important to consider their acceptability and the feasibility of delivery alongside opportunistic vaccination programmes for GBMSM, such as mpox and human papillomavirus (HPV).^22^ Given high uptake of HIV-PrEP in GBMSM, the impacts of changing care pathways and service resources must be thoroughly examined and informed by local needs prior to full-scale recommendations. Though long acting injectables are highly acceptable by GBMSM already taking HIV-PrEP, availability may not be enough to minimise unmet need due to individual-, system-, and policy-level barriers.

### Conclusion

As routine HIV-PrEP delivery continues across England, it is crucial to identify and address new and persistent barriers to access. Ensuring equitable uptake among those who can benefit requires a clear understanding of these challenges to facilitate informed decision-making for individual HIV-PrEP needs. HIV-PrEP monitoring and evaluation must continue to prioritise these aspects to improve access and delivery, and to help inform the potential role of emerging modalities, such as long acting injectables. Equally, the role of long acting injectables in modern service delivery will need to be assessed to minimise adverse harms and ensure informed decision-making for HIV-PrEP related needs.

## Data Availability

The data that support the findings of this study are not publicly available to protect participant privacy. However, some aggregate data are available upon reasonable request from the UK Health Security Agency (UKHSA). Requests can be directed to DataAccess@ukhsa.gov.uk.
